# 5-MeO-DALT: the freebase of *N*,*N*-diallyl-5-meth­oxy­tryptamine

**DOI:** 10.1107/S2414314620004988

**Published:** 2020-04-17

**Authors:** Andrew R. Chadeayne, Duyen N. K. Pham, James A. Golen, David R. Manke

**Affiliations:** a CaaMTech, LLC, 58 East Sunset Way, Suite 209, Issaquah, WA 98027, USA; b University of Massachusetts Dartmouth, 285 Old Westport Road, North Dartmouth, MA 02747, USA; University of Antofagasta, Chile

**Keywords:** crystal structure, tryptamines, indoles, hydrogen bonding

## Abstract

The title compound {systematic name: *N*-[2-(5-meth­oxy-1*H*-indol-3-yl)eth­yl]-*N*-(prop-2-en-1-yl)prop-2-en-1-amine), C_17_H_22_N_2_O, has a single tryptamine mol­ecule in the asymmetric unit. The mol­ecules are linked by strong N—H⋯N hydrogen bonds into zigzag chains with graph-set notation *C*(7) along the [010] direction.

## Abstract 2

The solid-state structure of the synthetic psychedelic 5-meth­oxy-*N*,*N*-di­allyl­tryptamine (5-MeO-DMT) is reported in its freebase form.

## Graphical abstract

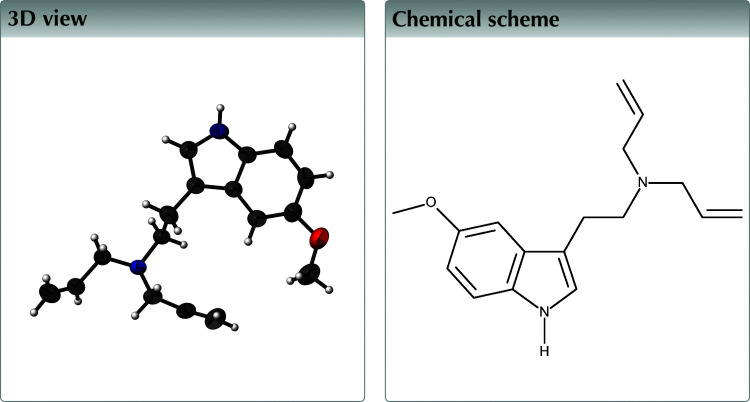

## Structure description

Psychedelics have garnered a great deal of study of late as potential therapeutics for mood disorders (Davis *et al.*, 2020 ▸; Carhart-Harris & Goodwin, 2017 ▸). Toads in the *Bufonidae* family release alkaloid secretions when they experience stress. These toads are the genesis of the urban myth of ‘licking toads’ because the secretion contains psychedelic tryptamines. The secretion has contents that can vary greatly from species to species. It is a medley of different chemicals; the skin of the species *Bufo alvarius*, a desert toad of Arizona, contains a number of indole­alkyl­amines, including bufotenine, *O*-methyl­bufotenine, and bufoviridine, among many others (Erspamer *et al.*, 1967 ▸).

Recent studies have shown that the psychotropic experiences of inhaling dried toad excretion and that of inhaling pure synthetic *O*-methyl­bufotenine [5-meth­oxy-*N*,*N*-di­methyl­tryptamine (5-MeO-DMT)] are markedly different (Uthaug, Lancelotta, van Oorsouw *et al.*, 2019 ▸; Uthaug, Lancelotta, Szabo *et al.*, 2019 ▸). The varied experiences suggests that the other tryptamines have significant activity in the psychedelic effects, or that they work in combination through an entourage effect. Accordingly, it is important to understand the pharmacology of not just 5-MeO-DMT, but all of the tryptamines in bufotoxin, and other related mol­ecules.

5-meth­oxy-*N*,*N*-di­allyl­tryptamine (5-MeO-DALT), streetname Foxtrot, is a synthetic analog of *O*-methyl­bufotenine first synthesized by Alexander Shulgin in 2004 (Shulgin & Shulgin, 2016 ▸). The compound is noted for its quick onset and rapid drop-off, when compared to other psychotropic tryptamines (Corkery *et al.*, 2012 ▸), and can cause acute delerium and rhabdomyolysis (Kalasho & Nielsen, 2016 ▸). The pharmacology of the compound demonstrates activity at the 5-hy­droxy­tryptamine (5-HT) receptors, particularly 5-HT_1A_, 5-HT_1D_, 5-HT_2B_, 5-HT_6_, and 5-HT_7_, though slightly less active at the 5-HT_2A_ receptor, which is believed to be responsible for most psychotropic activity (Cozzi & Daley, 2016 ▸). As these mol­ecules become more relevant in the treatment of mood disorders, it will be important to have analytically pure, well-characterized compounds, ideally as crystalline materials. Herein, we report the solid-state structure of 5-meth­oxy-*N*,*N*-di­allyl­tryptamine.

**Figure 1:**
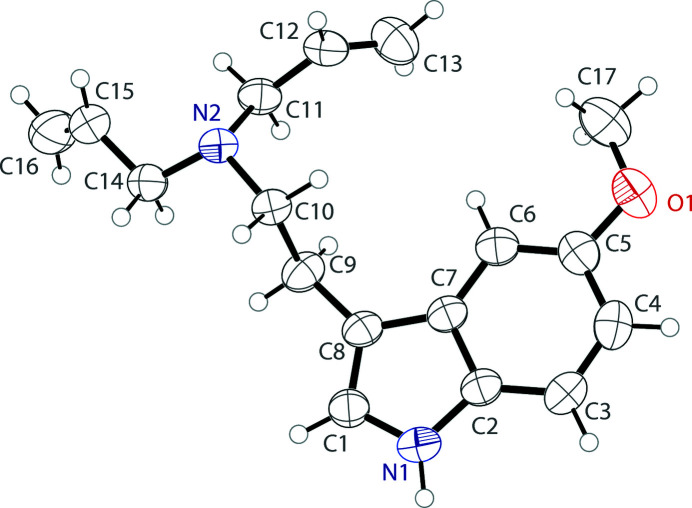
The mol­ecular structure of 5-meth­oxy-*N*,*N*-di­allyl­tryptamine, showing the atom labeling. Displacement ellipsoids are drawn at the 50% probability level.

**Figure 2:**
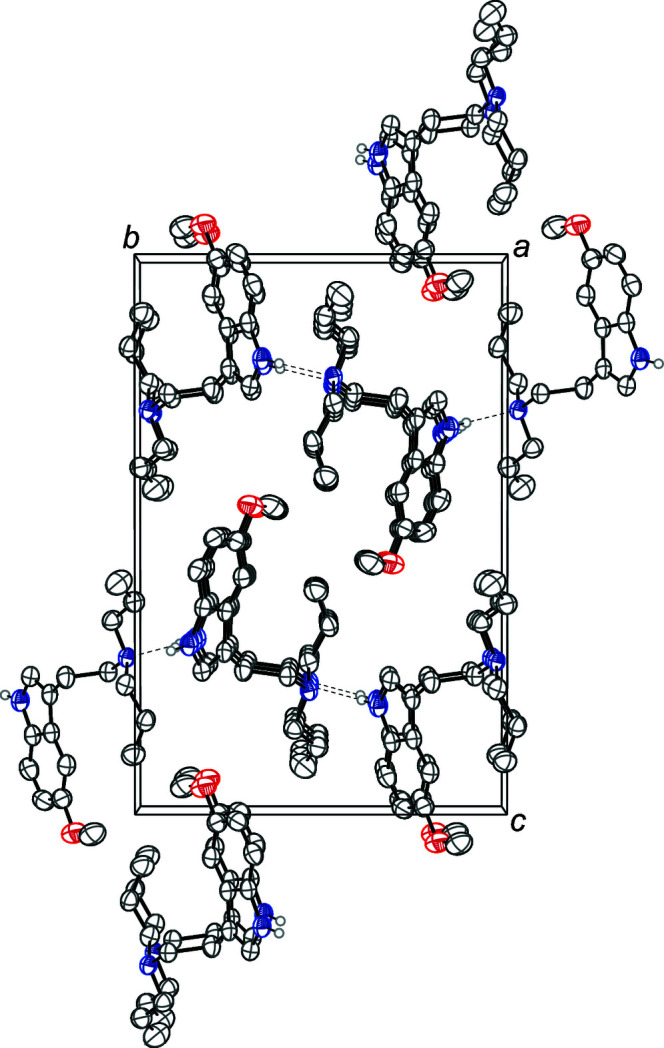
The crystal packing of 5-meth­oxy-*N*,*N*-di­allyl­tryptamine, viewed along the *a* axis. The N—H⋯N hydrogen bonds (Table 1 ▸) are shown as dashed lines. Displacement ellipsoids are drawn at the 50% probability level. Hydrogen atoms not involved in hydrogen bonding are omitted for clarity.

The asymmetric unit of 5-meth­oxy-*N*,*N*-di­allyl­tryptamine contains a single tryptamine mol­ecule (Fig. 1 ▸). The indole unit is nearly planar with a deviation from planarity of 0.015 Å. The meth­oxy group is in the same plane, with the indole and meth­oxy group showing an r.m.s. deviation of only 0.025 Å. The ethyl­amine group is turned significantly from the indole plane, with a C1—C8—C9—C10 torsion angle of 103.7 (2)°. The mol­ecules are held together by an N1—H1⋯N2 hydrogen bond between the indole N—H and the amino nitro­gen atom. These hydrogen bonds join the mol­ecules together along [010] (Table 1 ▸). The crystal packing of the title compound is shown in Fig. 2 ▸.

The title compound is similar to that of other 5-*O*-substituted tryptamines whose structures have been reported, including bufotenine (BUFTEN: Falkenberg, 1972*a* ▸), melatonin (MELATN: Wakahara *et al.*, 1972 ▸), 5-MeO-DMT hydro­chloride (MOTYPT: Falkenberg & Carlström, 1971 ▸), 5-meth­oxy­tryptamine (MXTRUP: Quarles *et al.*, 1974 ▸), 5-MeO-DMT and 5-meth­oxy­mono­methyl­tryptamine (QQQAGY & QQQAHA: Bergin *et al.*, 1968 ▸). The structure is also similar to the freebases of other psychedelic tryptamines that have been reported, including psilocybin (PSILOC: Weber & Petcher, 1974 ▸), psilocin (PSILIN: Petcher & Weber, 1974 ▸), *N*,*N*-di­methyl­tryptamine (DMTRYP: Falkenberg, 1972*b* ▸), *N*-methyl-*N*-propyl­tryptamine (WOHYAW: Chad­eayne, *et al.* 2019 ▸) and norpsilocin (Chadeayne *et al.*, 2020 ▸).

## Synthesis and crystallization

Slow evaporation of an acetone solution of a commercial sample (The Indole Shop) of 5-MeO-DALT freebase resulted in the formation of crystals of 5-meth­oxy-*N*,*N*-di­allyl­tryptamine suitable for X-ray analysis.

## Refinement

Crystal data, data collection and structure refinement details are summarized in Table 2 ▸.

## Supplementary Material

Crystal structure: contains datablock(s) I. DOI: 10.1107/S2414314620004988/bx4017sup1.cif

Structure factors: contains datablock(s) I. DOI: 10.1107/S2414314620004988/bx4017Isup2.hkl

CCDC reference: 1995802

Additional supporting information:  crystallographic information; 3D view; checkCIF report

## Figures and Tables

**Table 1:** Hydrogen-bond geometry (Å, °)

*D*—H⋯*A*	*D*—H	H⋯*A*	*D*⋯*A*	*D*—H⋯*A*
N1—H1⋯N2^i^	0.86 (1)	2.16 (1)	2.9880 (18)	162 (2)

Symmetry code: (i) 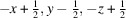 .

**Table 2:** Experimental details

Crystal data	
Chemical formula	C_17_H_22_N_2_O
*M*_r_	270.36
Crystal system, space group	Monoclinic, *P*2_1_/*n*
Temperature (K)	296
*a*, *b*, *c* (Å)	6.1444 (6), 12.8514 (13), 19.3315 (19)
β (°)	91.626 (3)
*V* (Å^3^)	1525.9 (3)
*Z*	4
Radiation type	Mo *K*α
μ (mm^−1^)	0.07
Crystal size (mm)	0.3 × 0.1 × 0.03

Data collection	
Diffractometer	Bruker D8 Venture CMOS
Absorption correction	Multi-scan (*SADABS*; Bruker, 2018 ▸)
*T*_min_, *T*_max_	0.694, 0.745
No. of measured, independent and observed [*I* > 2σ(*I*)] reflections	45375, 2810, 2145
*R*_int_	0.068
(sin θ/λ)_max_ (Å^−1^)	0.605

Refinement	
*R*[*F* ^2^ > 2σ(*F* ^2^)], *wR*(*F* ^2^), *S*	0.041, 0.106, 1.05
No. of reflections	2810
No. of parameters	186
No. of restraints	1
H-atom treatment	H atoms treated by a mixture of independent and constrained refinement
Δρ_max_, Δρ_min_ (e Å^−3^)	0.15, −0.13

Computer programs: *APEX3* and *SAINT* (Bruker, 2018 ▸), *SHELXT2014* (Sheldrick, 2015*a* ▸), *SHELXL2018* (Sheldrick, 2015*b* ▸), *OLEX2* (Dolomanov *et al.*, 2009 ▸) and *publCIF* (Westrip, 2010 ▸).

## full crystallographic data

### Crystal data

**Table:**

C_17_H_22_N_2_O	*F*(000) = 584
*M**_r_* = 270.36	*D*_x_ = 1.177 Mg m^−^^3^
Monoclinic, *P*2_1_/*n*	Mo *K*α radiation, λ = 0.71073 Å
*a* = 6.1444 (6) Å	Cell parameters from 7918 reflections
*b* = 12.8514 (13) Å	θ = 3.2–24.5°
*c* = 19.3315 (19) Å	µ = 0.07 mm^−^^1^
β = 91.626 (3)°	*T* = 296 K
*V* = 1525.9 (3) Å^3^	PLATE, colourless
*Z* = 4	0.3 × 0.1 × 0.03 mm

### Data collection

**Table:**

Bruker D8 Venture CMOS diffractometer	2145 reflections with *I* > 2σ(*I*)
φ and ω scans	*R*_int_ = 0.068
Absorption correction: multi-scan (SADABS; Bruker, 2018)	θ_max_ = 25.5°, θ_min_ = 3.2°
*T*_min_ = 0.694, *T*_max_ = 0.745	*h* = −7→7
45375 measured reflections	*k* = −15→15
2810 independent reflections	*l* = −23→23

### Refinement

**Table:**

Refinement on *F*^2^	Hydrogen site location: mixed
Least-squares matrix: full	H atoms treated by a mixture of independent and constrained refinement
*R*[*F*^2^ > 2σ(*F*^2^)] = 0.041	*w* = 1/[σ^2^(*F*_o_^2^) + (0.0421*P*)^2^ + 0.3975*P*] where *P* = (*F*_o_^2^ + 2*F*_c_^2^)/3
*wR*(*F*^2^) = 0.106	(Δ/σ)_max_ < 0.001
*S* = 1.05	Δρ_max_ = 0.15 e Å^−^^3^
2810 reflections	Δρ_min_ = −0.13 e Å^−^^3^
186 parameters	Extinction correction: SHELXL2018 (Sheldrick, 2015b), Fc^*^=kFc[1+0.001xFc^2^λ^3^/sin(2θ)]^-1/4^
1 restraint	Extinction coefficient: 0.036 (4)

### Special details

**Table:**

Geometry. All esds (except the esd in the dihedral angle between two l.s. planes) are estimated using the full covariance matrix. The cell esds are taken into account individually in the estimation of esds in distances, angles and torsion angles; correlations between esds in cell parameters are only used when they are defined by crystal symmetry. An approximate (isotropic) treatment of cell esds is used for estimating esds involving l.s. planes.
Refinement. Hydrogen atom H1 was found from a difference-Fourier map and refined isotropically, using a *DFIX* restrain with an N–H distance of 0.86 (1) Å. The isotropic displacement parameter was set to 1.2*U*_eq_ of the parent indolic nitrogen atom. All other hydrogen atoms were placed in calculated positions with appropriate carbon-hydrogen bond lengths: (*sp*^2^) 0.93 Å, (CH_3_) 0.96 Å, (CH_2_) 0.97 Å. Isotropic displacement parameters were set to 1.2*U*_eq_ (C) for *sp*^2^ and CH_2_ parent carbon atoms and 1.5*U*_eq_ (*C*-methyl)

### Fractional atomic coordinates and isotropic or equivalent isotropic displacement parameters (Å^2^)

**Table:**

	*x*	*y*	*z*	*U*_iso_*/*U*_eq_	
O1	0.4047 (2)	0.31452 (11)	0.55090 (6)	0.0675 (4)	
N1	0.0481 (2)	0.15647 (10)	0.30952 (7)	0.0470 (4)	
N2	0.71726 (19)	0.46740 (9)	0.22429 (6)	0.0382 (3)	
C1	0.1988 (3)	0.18855 (12)	0.26318 (9)	0.0459 (4)	
H1A	0.192685	0.173076	0.216165	0.055*	
C2	0.1120 (2)	0.19241 (11)	0.37388 (8)	0.0417 (4)	
C3	0.0196 (3)	0.17930 (13)	0.43823 (9)	0.0504 (4)	
H3	−0.109406	0.142352	0.442694	0.060*	
C4	0.1243 (3)	0.22242 (14)	0.49473 (9)	0.0541 (5)	
H4	0.064549	0.214786	0.538107	0.065*	
C5	0.3194 (3)	0.27783 (13)	0.48877 (9)	0.0491 (4)	
C6	0.4115 (3)	0.29288 (12)	0.42553 (8)	0.0452 (4)	
H6	0.539720	0.330673	0.421748	0.054*	
C7	0.3063 (2)	0.24944 (11)	0.36668 (8)	0.0392 (4)	
C8	0.3590 (2)	0.24612 (11)	0.29497 (8)	0.0406 (4)	
C9	0.5518 (3)	0.29470 (12)	0.26205 (9)	0.0473 (4)	
H9A	0.572877	0.262073	0.217492	0.057*	
H9B	0.680614	0.281261	0.290827	0.057*	
C10	0.5280 (2)	0.41216 (11)	0.25165 (8)	0.0389 (4)	
H10A	0.404340	0.424296	0.220377	0.047*	
H10B	0.493855	0.443084	0.295809	0.047*	
C11	0.9078 (2)	0.46679 (13)	0.27223 (8)	0.0445 (4)	
H11A	1.029007	0.500425	0.250178	0.053*	
H11B	0.949342	0.395345	0.281998	0.053*	
C12	0.8647 (3)	0.52092 (13)	0.33844 (9)	0.0479 (4)	
H12	0.791552	0.584226	0.335903	0.058*	
C13	0.9215 (3)	0.48650 (16)	0.39976 (10)	0.0649 (5)	
H13A	0.994865	0.423490	0.404413	0.078*	
H13B	0.888570	0.524946	0.438859	0.078*	
C14	0.7732 (3)	0.42746 (13)	0.15593 (8)	0.0478 (4)	
H14A	0.643138	0.426748	0.126309	0.057*	
H14B	0.823752	0.356257	0.160826	0.057*	
C15	0.9444 (3)	0.49012 (14)	0.12192 (9)	0.0540 (5)	
H15	0.939074	0.562062	0.126783	0.065*	
C16	1.0998 (4)	0.45147 (19)	0.08615 (11)	0.0799 (7)	
H16A	1.110188	0.379848	0.080213	0.096*	
H16B	1.201163	0.495318	0.066365	0.096*	
C17	0.6043 (4)	0.36914 (19)	0.54980 (11)	0.0765 (6)	
H17A	0.641883	0.393335	0.595580	0.115*	
H17B	0.589963	0.427531	0.519037	0.115*	
H17C	0.716441	0.323651	0.534050	0.115*	
H1	−0.053 (3)	0.1114 (13)	0.3001 (11)	0.092*	

### Atomic displacement parameters (Å^2^)

**Table:**

	*U*^11^	*U*^22^	*U*^33^	*U*^12^	*U*^13^	*U*^23^
O1	0.0743 (9)	0.0789 (9)	0.0489 (8)	0.0010 (7)	−0.0049 (6)	−0.0074 (6)
N1	0.0463 (8)	0.0388 (8)	0.0558 (9)	−0.0055 (6)	−0.0007 (6)	−0.0011 (6)
N2	0.0355 (6)	0.0353 (7)	0.0437 (7)	−0.0011 (5)	0.0004 (5)	−0.0024 (5)
C1	0.0538 (9)	0.0367 (8)	0.0475 (9)	0.0008 (7)	0.0026 (7)	−0.0016 (7)
C2	0.0418 (8)	0.0321 (8)	0.0511 (9)	0.0035 (6)	0.0001 (7)	0.0027 (7)
C3	0.0470 (9)	0.0445 (9)	0.0600 (11)	0.0000 (7)	0.0077 (8)	0.0084 (8)
C4	0.0584 (11)	0.0562 (10)	0.0481 (10)	0.0097 (8)	0.0087 (8)	0.0089 (8)
C5	0.0527 (10)	0.0472 (9)	0.0470 (10)	0.0095 (8)	−0.0031 (8)	−0.0005 (7)
C6	0.0432 (8)	0.0380 (8)	0.0542 (10)	0.0029 (7)	−0.0008 (7)	−0.0010 (7)
C7	0.0394 (8)	0.0299 (7)	0.0482 (9)	0.0045 (6)	0.0011 (7)	0.0017 (6)
C8	0.0430 (8)	0.0284 (7)	0.0505 (9)	0.0037 (6)	0.0036 (7)	0.0001 (6)
C9	0.0470 (9)	0.0369 (9)	0.0586 (10)	0.0028 (7)	0.0113 (8)	0.0011 (7)
C10	0.0342 (8)	0.0364 (8)	0.0459 (9)	0.0010 (6)	0.0001 (6)	0.0000 (6)
C11	0.0359 (8)	0.0437 (9)	0.0537 (10)	−0.0013 (7)	−0.0023 (7)	−0.0043 (7)
C12	0.0481 (9)	0.0401 (9)	0.0552 (10)	−0.0033 (7)	−0.0054 (8)	−0.0054 (7)
C13	0.0702 (13)	0.0677 (12)	0.0559 (11)	0.0061 (10)	−0.0122 (9)	−0.0051 (9)
C14	0.0490 (9)	0.0482 (9)	0.0465 (9)	−0.0083 (7)	0.0035 (7)	−0.0048 (7)
C15	0.0577 (11)	0.0540 (10)	0.0505 (10)	−0.0133 (8)	0.0043 (8)	−0.0003 (8)
C16	0.0770 (14)	0.0871 (15)	0.0769 (14)	−0.0315 (12)	0.0275 (12)	−0.0193 (12)
C17	0.0753 (14)	0.0892 (16)	0.0639 (13)	−0.0017 (12)	−0.0156 (10)	−0.0125 (11)

### Geometric parameters (Å, º)

**Table:**

O1—C5	1.380 (2)	C9—H9B	0.9700
O1—C17	1.414 (2)	C9—C10	1.529 (2)
N1—C1	1.370 (2)	C10—H10A	0.9700
N1—C2	1.374 (2)	C10—H10B	0.9700
N1—H1	0.863 (5)	C11—H11A	0.9700
N2—C10	1.4735 (18)	C11—H11B	0.9700
N2—C11	1.4726 (19)	C11—C12	1.487 (2)
N2—C14	1.4677 (19)	C12—H12	0.9300
C1—H1A	0.9300	C12—C13	1.303 (2)
C1—C8	1.364 (2)	C13—H13A	0.9300
C2—C3	1.392 (2)	C13—H13B	0.9300
C2—C7	1.411 (2)	C14—H14A	0.9700
C3—H3	0.9300	C14—H14B	0.9700
C3—C4	1.369 (2)	C14—C15	1.492 (2)
C4—H4	0.9300	C15—H15	0.9300
C4—C5	1.402 (2)	C15—C16	1.294 (3)
C5—C6	1.375 (2)	C16—H16A	0.9300
C6—H6	0.9300	C16—H16B	0.9300
C6—C7	1.408 (2)	C17—H17A	0.9600
C7—C8	1.433 (2)	C17—H17B	0.9600
C8—C9	1.497 (2)	C17—H17C	0.9600
C9—H9A	0.9700		
			
C5—O1—C17	117.69 (15)	N2—C10—C9	116.70 (12)
C1—N1—C2	108.04 (13)	N2—C10—H10A	108.1
C1—N1—H1	123.8 (15)	N2—C10—H10B	108.1
C2—N1—H1	127.0 (15)	C9—C10—H10A	108.1
C11—N2—C10	113.15 (12)	C9—C10—H10B	108.1
C14—N2—C10	111.23 (12)	H10A—C10—H10B	107.3
C14—N2—C11	111.24 (12)	N2—C11—H11A	109.1
N1—C1—H1A	124.4	N2—C11—H11B	109.1
C8—C1—N1	111.17 (14)	N2—C11—C12	112.40 (13)
C8—C1—H1A	124.4	H11A—C11—H11B	107.9
N1—C2—C3	130.91 (15)	C12—C11—H11A	109.1
N1—C2—C7	107.83 (14)	C12—C11—H11B	109.1
C3—C2—C7	121.25 (15)	C11—C12—H12	117.5
C2—C3—H3	121.0	C13—C12—C11	125.04 (17)
C4—C3—C2	118.00 (16)	C13—C12—H12	117.5
C4—C3—H3	121.0	C12—C13—H13A	120.0
C3—C4—H4	119.2	C12—C13—H13B	120.0
C3—C4—C5	121.57 (16)	H13A—C13—H13B	120.0
C5—C4—H4	119.2	N2—C14—H14A	108.9
O1—C5—C4	113.98 (15)	N2—C14—H14B	108.9
C6—C5—O1	124.76 (16)	N2—C14—C15	113.21 (13)
C6—C5—C4	121.26 (16)	H14A—C14—H14B	107.7
C5—C6—H6	120.9	C15—C14—H14A	108.9
C5—C6—C7	118.12 (15)	C15—C14—H14B	108.9
C7—C6—H6	120.9	C14—C15—H15	117.7
C2—C7—C8	107.18 (13)	C16—C15—C14	124.64 (18)
C6—C7—C2	119.78 (14)	C16—C15—H15	117.7
C6—C7—C8	133.00 (14)	C15—C16—H16A	120.0
C1—C8—C7	105.77 (13)	C15—C16—H16B	120.0
C1—C8—C9	127.18 (15)	H16A—C16—H16B	120.0
C7—C8—C9	127.04 (14)	O1—C17—H17A	109.5
C8—C9—H9A	108.9	O1—C17—H17B	109.5
C8—C9—H9B	108.9	O1—C17—H17C	109.5
C8—C9—C10	113.19 (13)	H17A—C17—H17B	109.5
H9A—C9—H9B	107.8	H17A—C17—H17C	109.5
C10—C9—H9A	108.9	H17B—C17—H17C	109.5
C10—C9—H9B	108.9		
			
O1—C5—C6—C7	179.25 (14)	C3—C4—C5—C6	1.3 (3)
N1—C1—C8—C7	0.67 (17)	C4—C5—C6—C7	−1.1 (2)
N1—C1—C8—C9	179.97 (14)	C5—C6—C7—C2	0.0 (2)
N1—C2—C3—C4	178.16 (16)	C5—C6—C7—C8	−177.13 (15)
N1—C2—C7—C6	−178.18 (13)	C6—C7—C8—C1	177.21 (16)
N1—C2—C7—C8	−0.40 (16)	C6—C7—C8—C9	−2.1 (3)
N2—C11—C12—C13	135.82 (18)	C7—C2—C3—C4	−0.8 (2)
N2—C14—C15—C16	140.91 (19)	C7—C8—C9—C10	−77.2 (2)
C1—N1—C2—C3	−178.24 (16)	C8—C9—C10—N2	175.60 (13)
C1—N1—C2—C7	0.80 (17)	C10—N2—C11—C12	−62.30 (17)
C1—C8—C9—C10	103.67 (18)	C10—N2—C14—C15	172.28 (13)
C2—N1—C1—C8	−0.94 (18)	C11—N2—C10—C9	−65.85 (17)
C2—C3—C4—C5	−0.3 (2)	C11—N2—C14—C15	−60.60 (18)
C2—C7—C8—C1	−0.16 (16)	C14—N2—C10—C9	60.23 (18)
C2—C7—C8—C9	−179.46 (14)	C14—N2—C11—C12	171.63 (13)
C3—C2—C7—C6	1.0 (2)	C17—O1—C5—C4	178.50 (16)
C3—C2—C7—C8	178.75 (14)	C17—O1—C5—C6	−1.8 (2)
C3—C4—C5—O1	−178.99 (15)		

### Hydrogen-bond geometry (Å, º)

**Table:**

*D*—H···*A*	*D*—H	H···*A*	*D*···*A*	*D*—H···*A*
N1—H1···N2^i^	0.86 (1)	2.15 (1)	2.9880 (18)	162 (2)

Symmetry code: (i) −*x*+1/2, *y*−1/2, −*z*+1/2.
